# Structural, electronic and bioactive profiling of a 2′-hydroxychalcone–Ag composite: a DFT-supported biomedical study on anticancer, antioxidant and antibacterial activities

**DOI:** 10.1039/d5ra06439b

**Published:** 2026-02-16

**Authors:** Ayesha Latif Butt, Farhat Saira, Safeer Ahmed, Sumbal Tahir, Anila Iqbal, Asima Siddiqa, Kehkashan Mazhar, Syeda Sohaila Naz

**Affiliations:** a Department of Chemistry, Quaid-i-Azam University 45320 Islamabad Pakistan safeerad@qau.edu.pk; b Nanoscience and Technology Division, National Centre for Physics 44000 Islamabad Pakistan fsghaus@gmail.com; c Institute of Biomedical and Genetic Engineering (IBGE), KRL Hospital G-9 Islamabad Pakistan

## Abstract

Biocompatible and biologically active nanomaterials are currently in demand. This work presents the synthesis of the medicinally significant fluorophore 2′-hydroxychalcone and its subsequent composite with silver nanoparticles (chal–AgNPs) and their characterization using experimental and theoretical techniques and applications. The active organic functional groups present in the chalcone functioned as reducing and stabilizing agents in the composite with AgNPs by solution chemistry method. FESEM reveals spherically shaped and uniformly scattered nanoparticles with an average diameter of 70 nm. X-ray diffraction (XRD) patterns of chalcone-mediated AgNPs confirmed the existence of spherical AgNPs with a crystallite size of 46 nm in a face-centered cubic crystal structure. The surface plasmon resonance of AgNPs was observed at 493 nm in the absorption spectrum. The surface adsorption of chal–AgNPs was further validated by mixing pre-synthesized AgNPs prepared by sodium borohydride reduction with chalcone and real-time monitoring of the UV-visible spectrum. The ^1^H NMR and ^13^C NMR spectra of the reaction further elucidated the structure and formation of 2′-hydroxychalcone. Fluorescence quenching of the chalcone moiety by AgNPs was monitored by comparing the steady-state fluorescence spectra of chalcone alone and chal–AgNPs. Theoretical studies were performed on chalcone and its complexes using density functional theory/B3LYP calculations to gain insights into the quenching mechanism. Mulliken charge analysis estimated the polarity and showed that the hydroxyl oxygen in the chalcone is more nucleophilic than the carbonyl oxygen, indicating its stronger reducing ability. The computed binding energies for the chal–AgNP composite were positive, indicating its stability. From the computed molecular electrostatic potential (MEP) surfaces, significant charge transfer was observed for the chalcone–silver complexes, which further validated the experimental results of fluorescence quenching. An assessment of the global reactivity descriptors of chal–AgNPs indicated their softness and higher biological activity compared with that of the free chalcone. DFT calculations showed that the electron from the chalcone was transferred to the Ag center, and consequently, Ag^+^ ions were reduced to Ag^0^ in the composite formation. The synthesized chal–AgNP nanocomposite shows potent antibacterial, anticancer and antioxidant activities.

## Abstract 2

2′-Hydroxychalcone–Ag complex has multiple biological applications.

## Graphical abstract

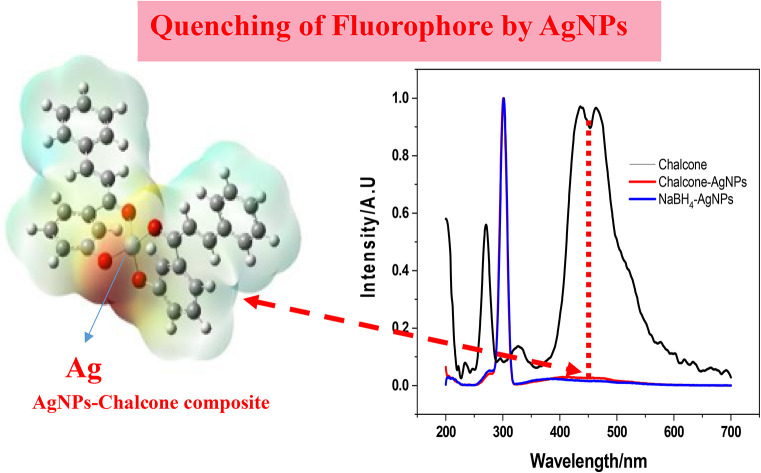

## 1. Introduction

Chalcones and their derivatives are small organic fluorophores which are found in a wide variety of molecular structures, some of which have been used as novel therapeutic compounds. They have potential applications as anticancer, antidiabetic, antifungal, anti-HIV, anti-inflammatory, antidiuretic, antioxidant, and antimalarial drugs.^1–8^ The keto-ethylenic (–COCH=CH–) group serves as the chromophore and the pharmacophore both for its biological activity, low toxicity and a wide range of possible substitutions which have made this moiety a privileged synthon that has been widely used in medicinal chemistry.

Nanoscale materials open up a world of possibilities in medicinal chemistry.^9–17^ Silver nanoparticles (AgNPs) have been one of the most investigated nanostructures over the last decade.^1,4,18,19^ Chemical reducing agents have been used to convert Ag ions to AgNPs using established synthesis methods.^19^ However, AgNPs obtained by applying these methods have biocompatibility issues, making their use unsuitable for biological systems. Biocompatible ligands are therefore employed to provide a buffer between the biological environment and nanoparticles.^1,4^ Chalcones, which are complexed with metals, have been shown to have improved catalytic properties in oxidation, hydrolysis, reduction, and many other processes.^20^ In a recent study, the antimalarial, cytotoxicity and haemolytic potentials of the chalcone were evaluated at the *in vitro* level using chalcone-conjugated AgNPs for enhanced performance.^1^ In another study, chalcones were used as emulsions for sunblock applications.^2^ Additionally, chalcones act as potent starting materials for many recent drug designs.^3^ The chalcones and their complexes are not just limited to pharmaceuticals but are also found in non-linear optics, polymers and corrosion inhibition, acting as a core of materials for these applications.^5^ The chalcones have alternate double and single bonds, as well as a delocalized-electron system, which results in lower redox potential values and, as a result, easier electron transfer (ET) reactions. They can form metal complexes in which one or more donor atoms are present in the vicinity of the carbonyl group. Since coordination reduces electron density, the chalcones can act as effective reducing agents for converting metal ions into metal nanoparticles. In this study, the potential of 2′-hydroxychalcone in the synthesis of nanoparticles is highlighted, and this is derived from the chalcone's ability to interact efficiently with metal ions, resulting in chelation and the reduction of the metal ions.^18–20^

Based on these observations, chalcone-mediated AgNPs were synthesized and characterized using various spectroscopic techniques,^21–30^ including Fourier transform infrared (FTIR), ultraviolet-visible (UV-vis), proton and carbon nuclear magnetic resonance (^1^H NMR, ^13^C NMR), and fluorescence spectroscopy. To augment the experimental results, a density functional theory (DFT) study was employed to categorize the probable contributing functionalities of the chalcone molecule to obtain insight into the experimental process of the AgNP synthesis.^31–44^ Experimental research was supported by a rigorous theoretical examination to establish the mechanism of electron transfer between the complexes. The DFT study helped obtain a theoretical interpretation of the electronic and molecular structure of the chalcone as well as its binding ability with silver ions. The relative energies of several intermediates were estimated and assessed, starting with Ag(chalcone)_2_, as well as the effect of substituting one chalcone molecule with a methanol molecule. Density functional theory (DFT) calculations showed that the electron from the chalcone is transferred to the Ag center; consequently, Ag^1+^ ions are reduced to Ag^0^ because of composite formation. The DPPH scavenging experiment was used to assess the radical scavenging impact of 2′-hydroxychalcone, chalcone-mediated and NaBH_4_-reduced AgNPs. These nanocomposites were applied against the human breast cancer (MCF-7) cell line for antitumor evaluation and were found to be potent.^45^ This is the first detailed DFT study of a chalcone–Ag complex and utilizes the findings in cell lines, antibacterial activities and antioxidant studies.

## 2. Experimental

### 2.1 Materials

2-Hydroxybenzaldehyde (Sigma-Aldrich, 98%), acetophenone (Sigma-Aldrich, 98%), potassium hydroxide (Sigma-Aldrich, ≥85%), 37% hydrochloric acid (Merck), and silver nitrate (Sigma-Aldrich, ≥99.0%) were used as purchased. The solvents used were methanol (Merck, 37%), ammonia solution (Merck, 25%), and deionized water.

### 2.2 Instrumentation

The absorption spectra were recorded in the 200–800 nm range using a JASCO (Tokyo, Japan) UV-visible spectrophotometer. ^1^H NMR and ^13^C NMR were recorded using a Bruker 300 MHz spectrometer with CDCl_3_ as a solvent and tetramethylsilane (TMS) as an internal standard. Chemical changes were estimated in parts per million (ppm). A field-emission scanning electron microscope (ZEISS Sigma 500VP) was used to acquire FESEM morphological images. FTIR spectra were recorded in the range of 4000–400 cm^−1^ using a Bruker Alpha-T spectrophotometer. The X-ray diffraction patterns of the chalcone, chalcone-mediated and sodium borohydride-reduced AgNPs were recorded at 25 °C using a Philips PW-1840 diffractometer with CuK(α) radiation in the range of 20–70°. A PerkinElmer LS 55 Fluorescence spectrometer was used to record the steady state fluorescence spectra of the chalcone, chalcone-mediated AgNPs and NaBH_4_-reduced AgNPs.

### 2.3 Synthesis

#### 2.3.1 Synthesis of 2′-hydroxychalcone

**Scheme 1:**
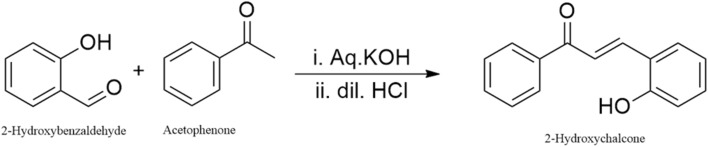
Synthesis scheme of 2′-hydroxychalcone.

The synthesis of 2′-hydroxychalcone is illustrated in Scheme 1. A Claisen–Schmidt condensation between 2′-hydroxybenzaldehyde and acetophenone was conducted using KOH (60%) as a catalyst. Briefly, equimolar quantities of acetophenone and 2′-hydroxybenzaldehyde were dissolved in methanol. Gradually, 10 mL of potassium hydroxide solution was added to the above mixture, and the reaction mixture was stirred overnight to ensure complete condensation. Upon completion, the mixture was poured slowly onto crushed ice, leading to the formation of a precipitate. The reaction medium was then acidified by the gradual addition of 10% aqueous hydrochloric acid (HCl) until the color of the mixture changed to yellow, indicating neutralization and product formation. The resulting suspension was allowed to stand to ensure complete precipitation. The solid product obtained was collected by vacuum filtration and thoroughly washed with cold distilled water to remove residual alkali and impurities. The crude product was air-dried and subsequently purified by recrystallization from 95% methanol, yielding pure 2′-hydroxychalcone as yellow crystalline solids.

The synthesized 2′-hydroxychalcone was structurally characterized and confirmed using Fourier-transform infrared spectroscopy (FTIR), proton nuclear magnetic resonance (^1^H NMR), carbon-13 nuclear magnetic resonance (^13^C NMR), and UV-visible spectroscopic techniques.

#### 2.3.2 Synthesis of the chalcone-mediated silver nanoparticles

An alcoholic solution of 2′-hydroxychalcone (5 mM) was mixed with an alcoholic solution of AgNO_3_ (0.25 mM) under ambient conditions. The pH of the reaction mixture was carefully adjusted to strongly basic conditions (pH 10–11) by the dropwise addition of an aqueous ammonia solution. The resulting homogeneous mixture was transferred to a round-bottom flask and subjected to reflux at 70 °C for 5–6 h under unstirred conditions. During the course of the reaction, a gradual change in the appearance of the solution was observed, indicating the formation of silver nanoparticles.

Upon completion of reflux, the reaction mixture was allowed to cool naturally to room temperature and subsequently left undisturbed overnight to ensure complete reduction and stabilization of the nanoparticles. A visible settled suspension of silver nanoparticles (AgNPs) was obtained. The hydroxyl functional group present at the 2′-position of the hydroxychalcone plays a crucial dual role in the synthesis, acting both as a reducing agent to convert Ag^+^ ions into metallic Ag^0^ and as a stabilizing (capping) agent to prevent excessive aggregation of the resulting nanoparticles. The reduced silver atoms subsequently nucleate and undergo controlled growth, leading to the formation of stable AgNPs. The synthesized AgNPs were isolated by centrifugation at 4000 rpm for 30 min and washed thoroughly with distilled water at least twice to remove unreacted precursors and residual ammonia. The purified nanoparticles were then dried under ambient conditions and stored for further physicochemical characterization.

### 2.4 Statistical analysis

All results are expressed as the mean ± standard deviation of three independent experiments. Statistical significance (*i.e.*, *p*-value) was calculated using a *t* test calculator (GraphPad Prism 10.1.1), and the data are considered statistically significant (indicated by *) when *p* < 0.05.

### 2.5 Computational study

Geometric optimization of free 2′-hydroxychalcone and its complexes with silver was carried out using the GAUSSIAN 09W program. The calculations used the 6-311G basis set for 2′-hydroxychalcone and LANL2DZ (Los Alamos National Laboratory 2 double-*ζ*) for the complexes. The vibrational frequency and optimized geometry of the observed molecules, as well as the highest occupied molecular orbital (HOMO) and lowest unoccupied molecular orbital (LUMO) molecular orbitals and the molecular electrostatic potential (MEP) surfaces, were pictured using the Becke, 3-parameter, Lee–Yang–Parr (B3LYP) basis set. A hybrid DFT methodology is used in the B3LYP framework. Amongst this growing variety of DFT techniques, the hybrid functional B3LYP provides good agreement between computing cost, range, and finding accuracy.^31–44^

## 3. Results and discussion

**Fig. 1:**
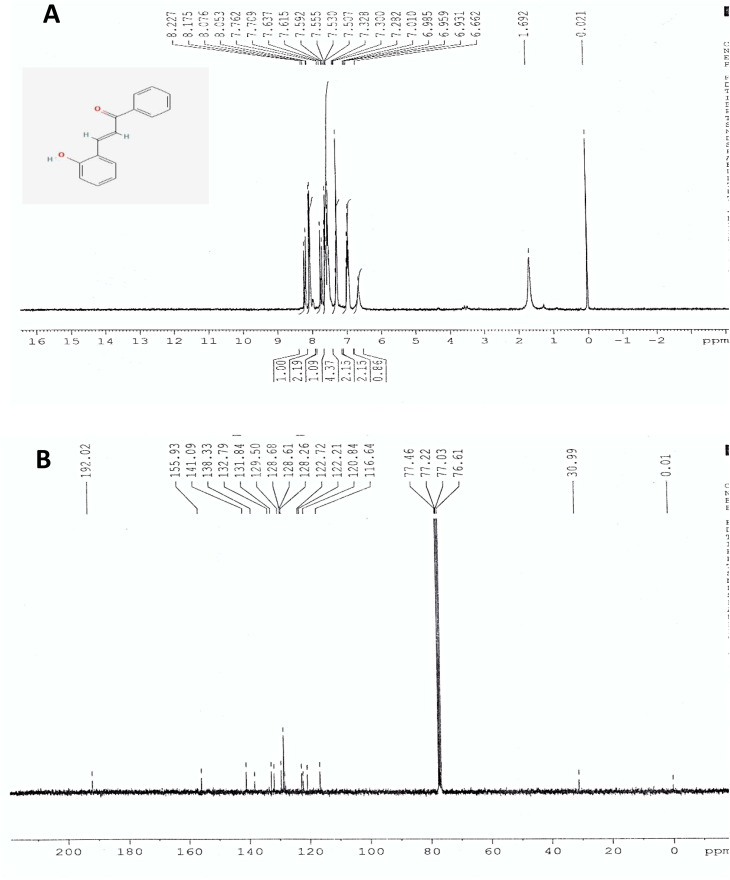
^1^H NMR (A) and ^13^C NMR (B) spectra of 2′-hydroxychalcone in deuterated acetone.

^1^H NMR (600 MHz, CDCl_3_) *δ*: 7.70 (d, *J* = 15.6 Hz, 1H, Hα), 7.53 (d, *J* = 15.6 Hz, 1H, Hβ), 7.90–7.10 (m, 9H, Ar–H), 9.85 (br s, 1H, OH). ^13^C NMR (150 MHz, CDCl_3_) *δ*: 192.15 (C=O), 157.02 (C–OH), 139.20, 132.64, 131.52 (Cα, Cβ, quaternary aromatic C), 129.72, 128.86, 128.34, 127.12, 126.65, 121.80 (aromatic CH), 115.98 (vinylic CH), 77.46, 77.22, 76.61 (CDCl_3_), 0.01 (TMS). The trans coupling constant (*J* = 15.6 Hz) between the α,β-olefinic protons confirms the *E*-configuration of the enone system. The phenolic OH resonance at *δ* 9.85 ppm and the corresponding C–OH signal at *δ* 157.0 ppm indicate that the hydroxyl group is located on the β-phenyl ring rather than adjacent to the carbonyl carbon. Spectral data are consistent with the literature values for 2′-hydroxychalcone. In detail, the ^1^H NMR spectrum (Fig. 1A) indicates the synthesis of 2′-hydroxychalcone and shows a strong peak of the solvent acetone at 2 ppm.^21^ The group of peaks from 6.92 to 7.92 represents the aromatic hydrogens, and they have a total integration assigned as 7 hydrogens. Peaks from 8.10–8.22 represent α and β hydrogens and have an integration of two. The peak at 9.2 correlates with the 2′-hydroxy hydrogen. Other peaks in the spectra include a peak at 3 ppm, which is probably a trace impurity from either the solvent or the sample. ^13^C NMR spectrum (Fig. 1B) displayed fifteen distinct carbon resonances, consistent with the proposed structure of 2′-hydroxychalcone. The ^13^C NMR spectrum of 2′-hydroxychalcone shows a distinct carbonyl carbon at *δ* 192.0 ppm, which is characteristic of a conjugated α,β-unsaturated ketone. The phenolic carbon (C–OH) resonates at *δ* 155.8 ppm, confirming hydroxyl substitution on the aromatic ring. The α- and β-carbons of the enone appear at *δ* 138.3 and 141.0 ppm, respectively, indicating extended conjugation. Multiple aromatic signals between *δ* 116.6 and 132.8 ppm correspond to substituted phenyl rings. The overall pattern supports the expected planar conjugated structure stabilized by intramolecular hydrogen bonding between the –OH and C=O groups.^21^

**Fig. 2:**
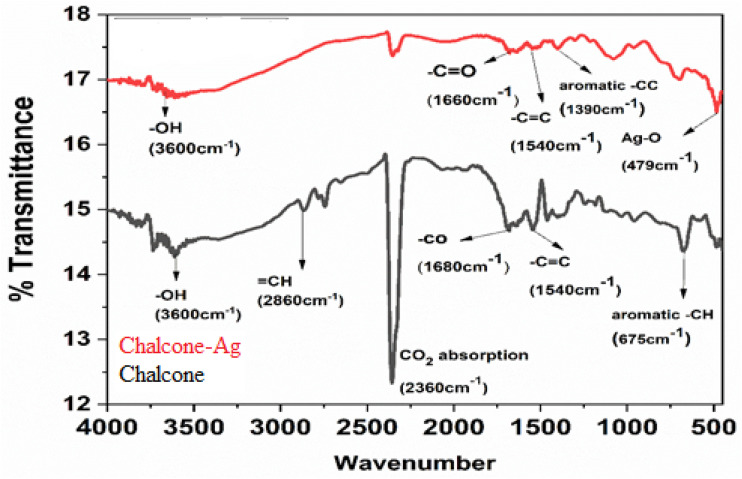
FTIR spectrum of 2′-hydroxychalcone and the chalcone-mediated AgNPs.

**Fig. 3:**
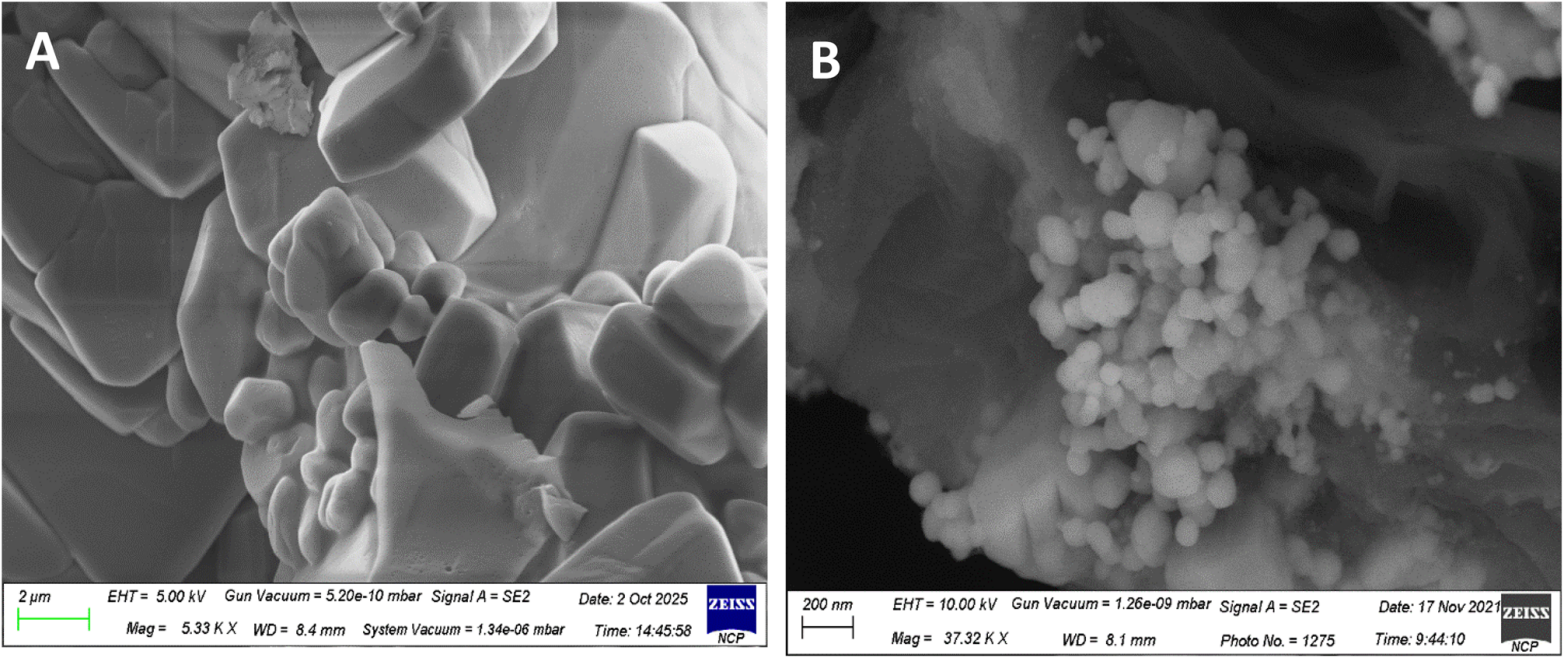
FESEM images of chalcone (A) and the chalcone–Ag nanocomposite (B).

The synthesis of 2′-hydroxychalcone was further established using FTIR spectroscopy.^21–24^ The identification using FTIR (Fig. 2) indicated absorption of the hydroxy bond at 3600 cm^−1^ (–OH). A conjugated double bond to a carbonyl group is distinguished by absorption emerging in the region of 1680 cm^−1^. The presence of the 1540 cm^−1^ absorption band confirms the existence of C=C aromatic. As in the case of the chalcone-mediated AgNPs, there is a weak band at 2350 cm^−1^ (atmospheric CO_2_). The C=O stretching band observed at 1660 cm^−1^ shifted from ∼1680 cm^−1^ for pure 2′-hydroxychalcone, indicating coordination of the carbonyl oxygen with Ag nanoparticles. The band at 1390 cm^−1^ corresponds to aromatic skeletal/C–H deformation influenced by nanoparticle interaction, while 1082 cm^−1^ is assigned to C–O stretching and possible C–O–Ag interactions, supporting the chalcone's role as a capping agent. The band at 479 cm^−1^ is consistent with Ag–O/metal–ligand vibrations, confirming the successful formation of the chalcone–AgNP composite.^23^ FESEM analysis reveals the chalcone particles in micron size and slightly agglomerated. Fig. 3A and spherical shape uniformly scattered nanoparticles of average 70 nm diameter for the chalcone-mediated AgNPs (Fig. 3B).

**Fig. 4:**
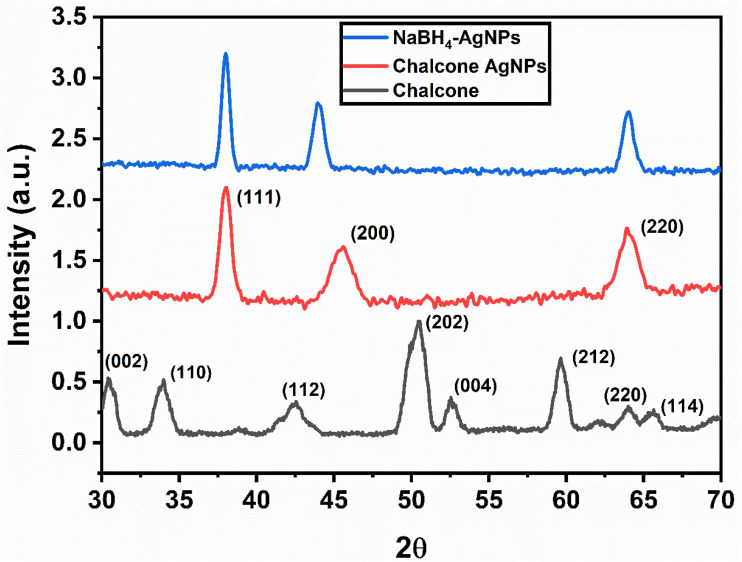
XRD diffraction patterns of chalcone and the chalcone-mediated AgNPs and NaBH_4_-reduced AgNPs matching with JCPDs: 1100136.

XRD analysis was used to determine the phase composition, purity, and crystal nature of the synthesized AgNPs.^24^ XRD patterns of the 2′-hydroxychalcone, chalcone-mediated and NaBH_4_-reduced AgNPs are displayed in Fig. 4. The Scherer equation was used to calculate the size of the AgNP crystallites. The XRD pattern of 2′-hydroxychalcone exhibits broad and low-intensity peaks at 2*θ* = 30.4°, 33.9°, 42.4°, 50.6°, 52.6°, 59.6°, 63.9°, and 65.6°, indicating its partially crystalline or semi-amorphous nature. The broadening of peaks suggests a small crystallite size and limited long-range molecular order, which is typical for organic compounds containing conjugated aromatic systems. Despite the reduced sharpness, the presence of distinct peaks confirms that the chalcone retains a crystalline molecular framework consistent with previously reported chalcone structures.^21^ The chalcone-mediated and NaBH_4_-reduced AgNPs showed diffraction peaks at 38.05, 44.24, and 64.47 and 37.95°, 44.09°, and 64.35°, respectively, which were indexed to the (111), (200), and (220) reflection planes of the face-centered cubic structure of metallic AgNPs.^22^ The lattice parameters of both the chalcone-mediated and NaBH_4_-reduced AgNPs were well matched with the crystallography open database JCPD: 1100136.

The X-ray diffraction peaks of the chalcone-mediated and NaBH_4_-reduced AgNPs differed in terms of intensity and broadening. The diffraction peaks of the NaBH_4_-reduced AgNPs are broadened, whereas the peaks of the chalcone-mediated nanoparticles are comparatively sharp. The development of small-sized silver nanoparticles is indicated by the broadening of the Bragg peaks,^21,24,25^ while the chalcone-mediated nanoparticles have sharp XRD peaks, suggesting relatively larger particles. The intensity of the XRD peaks of the chalcone-mediated AgNPs is less compared to NaBH_4_-reduced AgNPs. A prior study of nanoparticle's XRD data has shown an inverse relationship between nanoparticle surface functionalization and the intensity of the peak.^24^ As a consequence, the organic functional groups in 2′-hydroxychalcone capped the surface of AgNPs, resulting in reduced XRD peak intensity. This also indicates disruption of the chalcone crystallinity due to strong interaction and partial amorphousness caused by Ag nanoparticle formation.

**Fig. 5:**
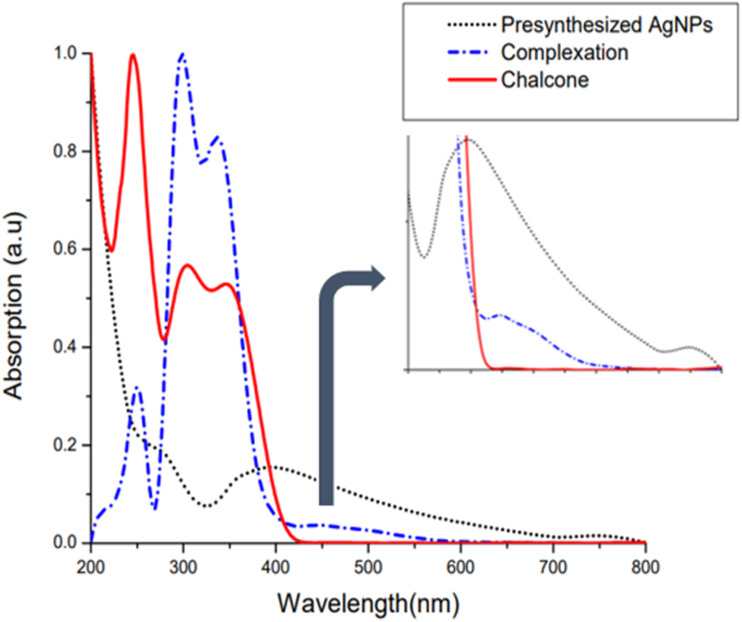
UV-visible spectroscopic analysis of chalcone, chalcone-mediated AgNPs and NaBH_4_-reduced AgNPs.

The 2′-hydroxychalcone-mediated AgNPs have a crystallite size of 46 nm, while that of NaBH_4_-reduced AgNPs is 24 nm, which is calculated using the Scherer equation. The synthesis of AgNPs was also examined by UV-vis spectroscopic analysis. The chalcone exhibited two main absorption bands in its UV-vis spectra. Band I was observed at a longer wavelength (near 300–390 nm) due to the absorption of the entire conjugated system, the cinnamoyl chromophore. Band II appeared at a shorter wavelength range of 220–270 nm. This specific band is attributed to the absorption involving the benzoyl chromophore, phenyl ring and adjacent carbonyl group. This band is primarily attributed to a π → π* electronic transition within the conjugated system (Table 1). The NaBH_4_-reduced AgNPs had a surface plasmon resonance (SPR) at 396 nm; however, the chalcone-mediated AgNPs showed an SPR at 493 nm, a difference of 100 nm towards a higher wavelength, as SPR is sensitive to the presence of the functional moieties.^25,26^ A prominent red shift in the SPR is also a confirmation for the binding of 2′-hydroxychalcone functional groups on the silver nanoparticle surface. In addition, in the case of the chalcone-mediated AgNPs, a band at a shorter wavelength (355 nm) is associated with the absorption of the 2′-hydroxychalcone as observed earlier in the pure 2′-hydroxychalcone spectrum, as depicted in Fig. 5. The combined impact of the chalcone capping, as well as the change in the surrounding shell of nanoparticles, might be responsible for such a red shift in the case of the chalcone-mediated AgNPs. This is also consistent with smaller particles having larger band gaps compared to larger particles. In addition, there is a possibility of band formation due to AgNPs and the chalcone interaction.

**Table 1:** UV-visible data of 2′-hydroxychalcone and the corresponding transitions

Bands	*λ*_max_ (nm)	Functional groups	Transition
Band I	340–390	Conjugated π system	π–π*
Band II	220–270	Benzoyl system	n–π*
Band III	300–320	(–CH=CH)	π–π*

This red shift indicates a larger size of particles, while the broadness of the absorption band is accompanied by a wider size distribution.^25,26^ The contribution of pure ionic silver in the near UV region (about 300 nm wavelength) is visible. The chalcone adsorption at the surface of AgNPs is evaluated by mixing NaBH_4_-reduced AgNPs with the chalcone and instantly measuring UV-visible spectra. Fig. 5 depicts the UV-visible profiles of the complexation of the chalcone and NaBH_4_-reduced AgNPs. A weak broad band around 446 nm is due to the adsorption of the chalcone on the NaBH_4_-reduced AgNP surface. The peak red-shifts are due to the complexation process between the chalcone and AgNPs. It diminishes the absorption band of 2′-hydroxychalcone at 243 nm, indicating that benzoyl (responsible for absorbance at this wavelength) triggers the adsorption of the chalcone on the nanoparticles.

**Fig. 6:**
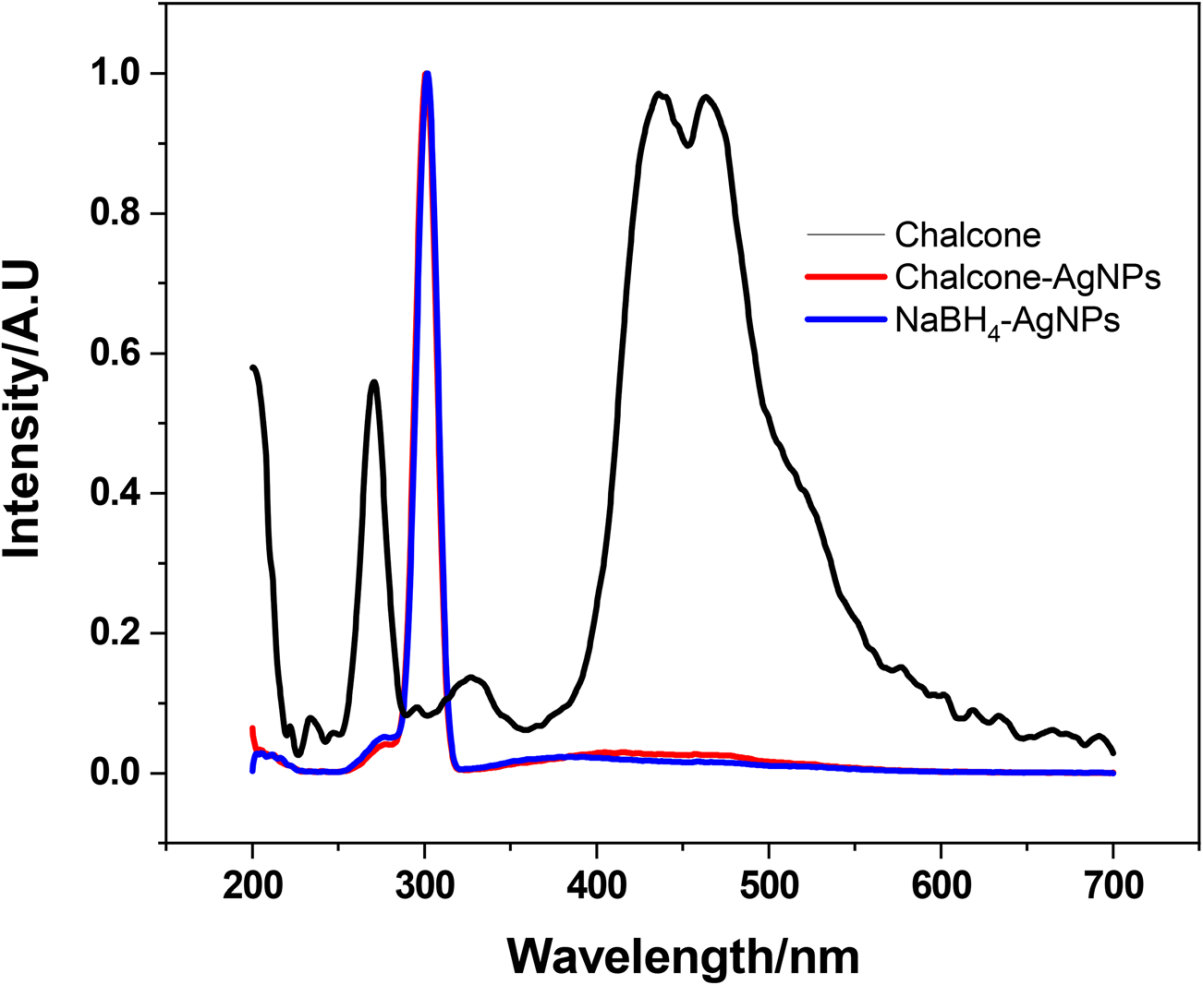
Fluorescence spectra of chalcone, chalcone-mediated AgNPs and NaBH_4_-reduced AgNPs.

Fluorescence spectroscopy was conducted to identify the complex formation of silver and chalcone during nanoparticle synthesis, as shown in Fig. 6. The fluorescence spectra of the chalcone and chalcone-reduced AgNPs were examined. When excitation occurs at 270 nm, the emission peaks of the chalcone in DMSO, which are evident at 435 and 466 nm, are missing in the AgNPs produced by the chalcone. The fluorescence of the chromophore in close vicinity to the metal nanoparticle's surface is well known to be highly impacted by the amplified electromagnetic field.^27–30^ The oxidized moieties of the chalcone on the AgNP surface interact with the surface electronically, which donates electrons to the metal, thereby quenching fluorescence. The fluorescence quenching in the presence of AgNPs gives a strong indication of the chalcone binding and capping with AgNPs.

When excited at the same excitation wavelength, the chalcone-mediated AgNPs showed more broadened absorption spectra at a longer wavelength (red shift) compared to NaBH_4_-reduced AgNPs, and the same reason is valid, as discussed above in UV-vis analysis.

### 3.1 Computational studies

#### 3.1.1 Geometry optimization

**Fig. 7:**
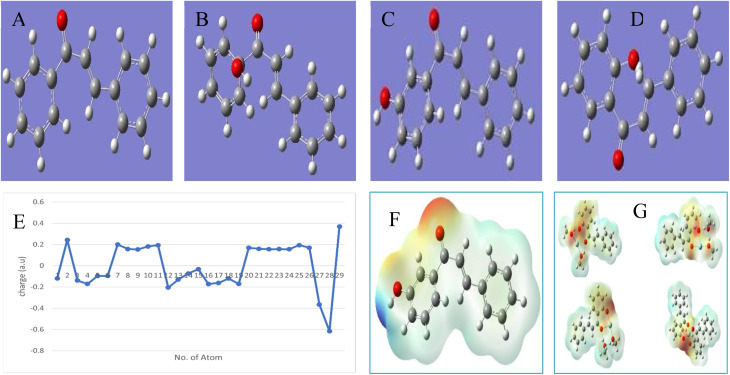
Geometry optimization of different hydroxyl derivatives of chalcone (A–D). MEP surface of 2′-hydroxychalcone (E). MEP surface of silver in the chalcone complexes (F). Graphical representation of charge distribution in 2′-hydroxychalcone (G).

From the geometry optimization of different hydroxyl derivatives of the chalcone, the minimum energy structures were obtained, as shown in Fig. 7A–D. Different hydroxyl derivatives of the chalcone are found to be more stable than the parent molecule and possess planar structures. Among the hydroxyl derivatives, 2′-hydroxychalcone is found to be the most stable, with −729.234 a.u. energy. Based on this calculation, 2′-hydroxychalcone was used for further investigation.

#### 3.1.2 Mulliken atomic charge population analysis

Mulliken population analysis is a technique for the calculation of partial atomic charges from computational chemistry studies, specifically based on the approach of LCAOs.^31^ Many quantum theoretical factors, such as dipole moment, polarization, and electronic structural features, are influenced by the charge distribution of donor and acceptor atoms in molecules.^32,33^ During the investigation of 2′-hydroxychalcone, the presence of an electrophilic and a nucleophilic atomic charge was observed (Fig. 7E). All the hydrogen atoms have an electrophilic charge (0.15–0.37 a.u.), and the two carbonyl carbon atoms have electrophilic behavior (0.19–0.24 a.u.). The nucleophilicity of all the ring carbon atoms was affected by the delocalization of phenyl electrons in the unsaturated and aromatic bonds in the chalcone backbone, which affected the nucleophilicity of all carbon atoms (−0.03–0.20 a.u.).

The study shows that the O atoms are strongly electronegative and have strong nucleophilicity values (−0.364–0.615 a.u.). The strong nucleophilic character of hydroxyl oxygen shows a stronger donating ability than the Mulliken atomic charge on each atom, as shown in Table S1.

#### 3.1.3 Interactions of the chalcone and silver ions

It is vital to explore all the different ways in which Ag^1+^ ions might interact with a molecule before comprehending the interaction between the chalcone and the Ag^1+^ ions. A B3LYP approach^39^ is used to study the complexes created by the probable interaction of a metal ion and 2′-hydroxychalcone. Fig. S1 shows the corresponding optimized structures.

Fig. S1(a) shows the configuration in which one silver atom is complexed with two 2′-hydroxychalcone molecules. The magnitude of the optimum energy changes when we mix a methanol molecule with a chalcone molecule to relate to the experimental solvent conditions (Fig. S1(b)). When the chalcone ligand is replaced by two methanol molecules, three structural patterns emerge for the bismethanol chalcone complex, Ag(2′-hydroxychalcone)(CH_3_OH)_2_. The very first configuration is one in which the methanol molecule links asymmetrically with the chalcone, and the second methanol molecule is linked to the first methanol molecule linearly through hydrogen bonding (Fig. S1(c)). The second configuration, in which a methanol molecule hydrogen bonds to the chalcone and the Ag atom is linked to the methanol molecule's O atom, is also possible (Fig. S1(d)). Fig. S1(e) depicts the third configuration, in which a methanol bridge is created between the Ag atom and one of the oxygen atoms of the chalcone's diketone portion. In addition to the complexes of the chalcones with Ag in the presence of methanol mentioned above, we computed complexes of the chalcones with three Ag^1+^ ions (Fig. 7F and G).

#### 3.1.4 Binding energies of 2′-hydroxychalcone–Ag complexes

The binding energies (B.E.) of different complexes of the chalcone with Ag are computed to compare the stability of the complexes, as listed in Table 2. The binding energy (B.E.) of a complex is defined as follows:

1 B.E. = *E*_complex_ − *E*_reactants_,

where *E*_complex_ is the energy of the chalcone complexes with Ag and *E*_reactants_ is the sum of energies of constituents of the complex. The binding energies (B.E.) of the complexes of the chalcone with Ag^1+^ ion were computed using the above-mentioned equation, and the values are tabulated (see Table 2). Thus, based on binding energy, the Ag(C)_2_-coordinated complex appears to be more stable in comparison to the other interactions although it seems that all the coordination complexes of the chalcone with silver are more or less probable.

**Table 2:** Optimization energies (*E*_optimization_), binding energies (B.E.), and input and output charges of silver ions

Complex	*E*_optimization_ (hartree)	B.E. (eV)	Charge input (a.u.)	Charge output (a.u.)
Ag(C)_2_	−1601.9985	60.84	+1	+0.444948
Ag(C)(M)	−990.0702	17.14	+1	+0.346315
Ag(C)(M)_2_	−1105.8234	16.37	+1	+0.293990
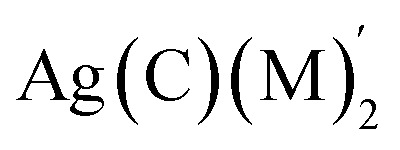	−1105.8078	16.78	+1	+0.354672
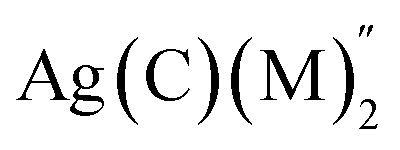	−1105.8199	16.45	+1	+0.274662
3Ag(C)	−1165.9584	15.00	+3	+0.436511

#### 3.1.5 2′-Hydroxychalcone as a reducing agent

As described in the experimental section, our findings showed that chalcone may reduce Ag^1+^ to Ag to synthesize AgNPs. However, it is difficult to demonstrate the involvement of the various moieties in the reduction and capping processes experimentally. We used molecular electrostatic potential (MEP) mapping to confirm and understand the reducing characteristics of the chalcones. The electrostatic potential is connected to the dipole moment, electronegativity, partial charge, and location of the molecule's chemical reaction.^32^ It provides a visual technique for understanding a molecule's relative polarity.

Fig. 7F and G depicts the electron density surface on which the electrostatic potential surface is mapped with the chalcone alone and its coordinate complex with Ag^1+^ ions, *i.e.*, Ag(chalcone)_2_ and Ag(chalcone)(methanol)_2_. Such surfaces reflect the size, shape, charge density, and position of the chemical interaction of the molecules.^36^ Varying colors reflect different degrees of electrostatic potential on the surface; red indicates regions with the greatest negative electrostatic potential, blue represents areas with the greatest positive electrostatic potential, and green indicates regions with zero potential. Potential rises in the following order: red > orange > yellow > green > blue.^36–38^

Because the O atom is electronegative in the chalcones, it has the largest negative electrostatic potential (red color). However, as shown in Fig. 7F and G, there was a significant degree of charge transfer in the Ag(chalcone)_2_ and Ag(chalcone)(methanol)_2_ complexes. Table 2 shows the numerical values of the charge decrease in Ag^1+^ ions in various chalcone compounds containing Ag atoms. The input charge on each Ag atom was specified as +1 in all complexes of the chalcones with silver atoms, which, after optimization, turned out to be a charge of roughly 0. Fig. 7G depicts the charge reduction diagrams on Ag^1+^ ions in various Ag(chalcone) complexes obtained from DFT calculations. One 2′-hydroxychalcone molecule can simultaneously reduce two Ag^1+^ ions.

#### 3.1.6 Global reactivity descriptors

It is critical to understand the chemical reactive characteristics of these silver–chalcone complexes before considering them as a starting point for the manufacture of drugs.^1^ Conceptual DFT is one of the most powerful tools in computational chemistry for understanding the chemical reactivity of interacting molecular systems, also known as chemical reactivity theory, because it allows for accomplishing this task by relying on a number of global reactivity descriptors (GRDs), which are related to variations in the electronic density of the systems studied.^38^ The characteristic values of HOMO and LUMO, as well as their energy band gap, are related to a molecule's biological activity.^33–36^ The molecule's ionization potential is directly linked to the HOMO energy, which indicates the electron donating potential. Meanwhile, the acceptance of electron affinity electrons is proportional to the degree of LUMO energy. DFT theoretical calculations were used to determine the compound's HOMO and LUMO energy levels.

The GRDs of the desired molecule, like electrophilicity (*ω*), hardness (*η*), electronegativity (*χ*), chemical potential (*µ*) and softness (*σ*) indexes, were calculated as quantum parameters from the HOMO/LUMO energy gap using Koopman's notation.^38^

In terms of *E*_HOMO_ and *E*_LUMO_, the HOMO energy and LUMO energy, respectively, ionization potential (I.P.) and electron affinity (E.A.) may be represented as follows:

2 I.P. = −*E*_HOMO_ and E.A. = −*E*_LUMO_.

When the values of I.P. and E.A. are known, the values of the electronegativity (*χ*), the hardness (*η*), the chemical potential (*µ*) and the softness (*σ*) can be computed using the following equations:

3 *χ* = I.P. + E.A./2, *η* = I.P. − E.A./2, *σ* = 1/*η*,

4 *ω* = *µ*^2^/2*η*.

This index, by definition, evaluates the ability of a chemical species to receive electrons. Low values of *µ* and *ω* characterize a good, highly reactive nucleophile, while high values of *µ* and *ω* characterize a good electrophile. This novel reactivity parameter quantifies the energy stabilization that occurs when the system receives an extra electronic charge *N*_max_ from the environment.

5 Δ*N*_max_ = −*µ*/*η*

Thus, while electron affinity indicates the system's tendency to acquire extra electronic charge from its surroundings, *N*_max_ indicates the molecule's charge potential.

The ligand acts as the Lewis base in a complex formation mechanism, while the metal ion acts as the Lewis acid. A ligand with an apt softness value has a high ability to bind metal ions. Table 3 displays the estimated quantum chemical descriptors, and the comparison research reveals the following issues about the free ligand: the ligand's soft character indicates its flexibility to metal atoms; negative electronic chemical potential and positive electrophilicity values signify that the molecule can capture electrons from its environment and that accepting an electronic charge should have a lower energy. As a result, it must have a negative electrical chemical potential.^35^

**Table 3:** Global reactivity descriptors for chalcone and its complexes with silver

	*E*_HOMO_ (eV)	*E*_LUMO_ (eV)	*E*_ap_ (eV)	*χ* (eV)	*η* (eV)	*σ* (eV^−1^)	*µ* (eV)	*ω* (eV)	Δ*N*_max_
C	−6.58	−2.35	4.23	4.46	2.11	0.47	−4.46	4.71	2.1
Ag(C)_2_	−5.48	−2.94	2.53	4.21	1.26	0.78	−4.21	6.98	3.3
Ag(C)(M)	−4.82	−1.85	2.97	3.34	1.48	0.67	−3.34	3.76	2.2
Ag(C)(M)_2_	−5.27	−2.28	2.98	3.78	1.49	0.67	−3.78	4.79	2.5
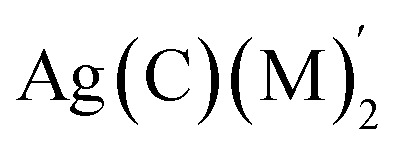	−5.09	−2.15	2.93	3.62	1.46	0.68	−3.6	4.46	2.4
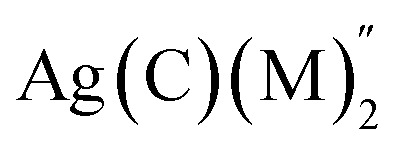	−5.10	−2.27	2.83	3.69	1.41	0.70	−3.69	4.80	2.6
3Ag(C)	−5.13	−2.81	2.32	3.97	1.16	0.86	−3.97	6.79	3.4

The following crucial points are introduced by quantum parameters^34,41,42^ for the apparent structures of metal complexes. A decrease in the energy gap compared to the free ligand reflects a higher softness of the complexes and thus an increase in their biological activity compared to the free chalcone. Greater values of electrophilicity and more negative values of chemical potential in complexes indicate that they can capture electrons more easily. The *E*_LUMO_ decreases and the *E*_HOMO_ increases more than the free chalcone, which can be attributed to the strength of the metal bonds.^41,42^

## 4. Biomedical applications of the chalcone–Ag nanoparticles

All the biomedical application results were expressed as the mean ± standard deviation of three independent experiments. Statistical significance (*i.e.*, *p*-value) was calculated using a *t* test calculator (GraphPad Prism 10.1.1), and the data are considered statistically significant (indicated by *) when *p* < 0.05.

The DPPH scavenging experiment was used to assess the radical scavenging impact of 2′-hydroxychalcone, the chalcone-mediated and NaBH_4_-reduced AgNPs. 2′-hydroxychalcone has more scavenging efficacy than the chalcone-mediated and NaBH_4_-reduced AgNPs. Due to the delayed release of active chalcone in the medium, the chalcone in the free form can quench free radicals more efficiently than the chalcone-mediated NPs. Although the DPPH assay is easy and basic, it has drawbacks. The substances that respond quickly to a peroxyl radical might react slowly or simply not with DPPH.^46^

As a result, the samples were further examined utilizing 2,2′-azinobis(3-ethylbenzothiazoline-6-sulphonic acid) (ABTS) assays. The ABTS analysis determines the efficiency with which antioxidants scavenge ABTS and produce ABTS radicals. Antioxidants act as hydrogen donors. The blue/green color of ABTS˙^+^ is caused by the interaction of ABTS with a strong oxidant, which results in the formation of radical cations.

**Fig. 8:**
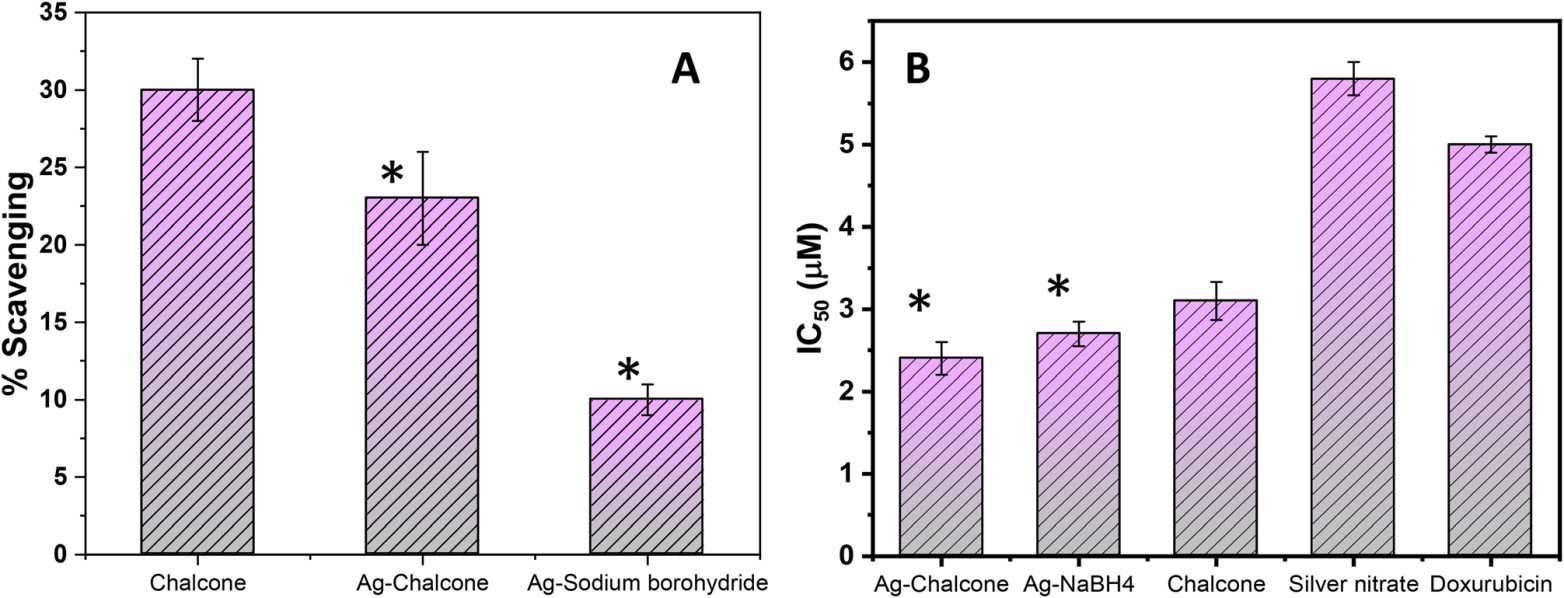
Radical scavenging effect of chalcone and the chalcone-mediated and NaBH_4_-reduced AgNPs by ABTS assay (A). *In vitro* anti-cancer activity of chalcone and the chalcone-mediated and NaBH_4_-reduced AgNPs along with AgNO_3_ and doxorubicin is taken as standard anticancer drug for comparison (B). Statistical significance (*i.e.*, *p*-value) was calculated using a *t* test calculator (GraphPad Prism 10.1.1), and the data are considered statistically significant (indicated by *) when *p* < 0.05.

The antioxidant activity determined by the ABTS radical scavenging analysis is depicted in Fig. 8A. The chalcone has an IC_50_ of 74.6 µg mL^−1^, which is lower than the chalcone-mediated and NaBH_4_-reduced AgNPs with IC_50_ values of 79.95 µg mL^−1^ and 120.61 µg mL^−1^, respectively. The antioxidant potential of the chalcone-mediated AgNPs is greater than that of NaBH_4_-reduced AgNPs.

AgNP antimicrobial properties have led to their widespread usage in the clinical and industrial fields. The chalcone-mediated and NaBH_4_-reduced AgNPs were shown to exhibit antibacterial action against Gram-positive and Gram-negative bacteria in this investigation. Table 4 shows the size of the zone of inhibition.

**Table 4:** Antibacterial activity of chalcone and the chalcone-mediated and NaBH_4_-reduced AgNPs

Bacteria	Zone of inhibition (mm)		
	2′-Hydroxychalcone	Ag–chalcone	Ag–NaBH_4_
*P. aeruginosa*	7 ± 1.2	0	0
*E. coli*	12 ± 2.5	8 ± 1.0	11 ± 0.8
*K. pneumoniae*	7 ± 0.8	0	7 ± 0.75
*S. aureus*	7 ± 1.5	7 ± 1.2	7 ± 0.5

After 24 hours of incubation, all the samples exhibited antibacterial activity, as evidenced by the presence of zones of inhibition.^20^ The findings of the disc diffusion procedure reveal that the greatest activity is displayed against *E. coli*. However, the chalcone and AgNPs synthesized through both routes had the same degree of activity against *S. aureus*, with zones of inhibition of 7 mm. No activity was noted against *P. aeruginosa* by the chalcone-mediated and NaBH_4_-reduced AgNPs. The chalcone-mediated AgNPs were inactive against *K. pneumoniae*, while the chalcone and NaBH_4_-reduced AgNPs showed the same inhibition zones.

As a result of the release of silver ions or the production of radical species after AgNPs are taken up by cells, AgNPs exhibit intrinsic anticancer characteristics. In the present study, the cytotoxic impact of the chalcone, chalcone-mediated, and NaBH_4_-reduced AgNPs against the human breast cancer (MCF-7) cell line and cytotoxicity (%) were measured *in vitro* using the MTT test and compared to the standard anticancer drug, doxorubicin. All the samples had concentrations ranging from 0.5 to 5 µg mL^−1^. The rate of cytotoxicity against the MCF-7 cell lines increases as the concentration increases. After 48 hours of incubation, the inhibition activity was observed. The results are shown in Fig. 8B as growth inhibitory concentration (IC_50_) values, which indicate the drug concentrations necessary to inhibit cell growth by 50% after 48 hours of incubation when compared with the untreated controls.

A synergistic effect was observed in the case of chalcone-mediated AgNPs for anticancer activity, as both the components have established individual anticancer activity. The silver salt also showed some anticancer activity, but no significant enhancement was observed compared with the synthesized AgNPs. Chalcone-mediated AgNPs are even more lethal to cancer cells than doxorubicin, a standard anticancer drug, and showed an IC_50_ value of 2.44 µM compared to doxorubicin, with an IC_50_ value of 5.01 µM.

**Fig. 9:**
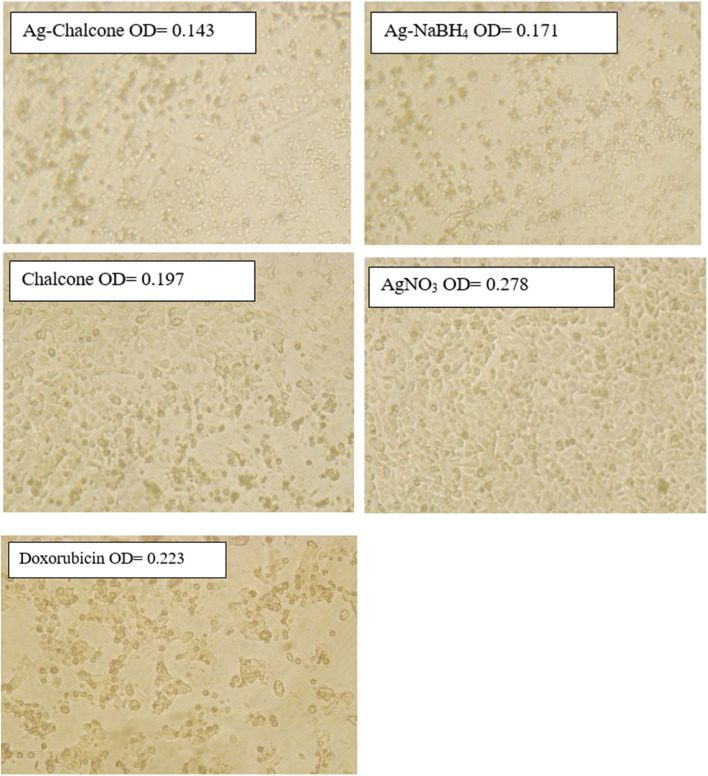
Morphological observation of the MCF-7 cancer cell lines using a bright field microscope.

A higher crystallization in microscopic images means higher cell death because the MTT assay's active component reacts with the cells and results in the form of crystallization. The optical density (OD) obtained for the different samples is well supported by the microscopic images. The higher OD value samples show lower crystallization in the images and *vice versa*, as illustrated in Fig. 9.

The chalcones are non-toxic and biocompatible capping agents; they can be considered environmentally friendly^46,47^ because they do not cause any environmental hazards, like those employed in the chemical reduction technique. Thus, the chalcone-mediated AgNPs have the potential to play a significant role in the advancement of possible nanomedicine as an antioxidant and against a variety of bacterial strains and potent cancer therapies.

## 5. Conclusion

In the present work, a highly facile, efficient, and environmentally benign synthetic route for silver nanoparticles (AgNPs) is successfully developed using 2′-hydroxychalcone as both a reducing and stabilizing (capping) agent. Multi-spectroscopic characterization techniques conclusively confirmed the effective capping of AgNPs by chalcone molecules, indicating strong interactions between the organic ligand and the metallic surface. The adsorption mechanism of 2′-hydroxychalcone on the AgNP surface was further validated through comparative studies using pre-synthesized sodium borohydride-reduced AgNPs, which provided clear evidence of chalcone-mediated surface functionalization. Density functional theory (DFT) calculations revealed a pronounced electron transfer from the 2′-hydroxychalcone molecule to Ag^+^ ions, leading to their reduction into metallic Ag^0^ through complex formation. Moreover, the optimized chalcone–metal complexes exhibited enhanced softness and reactivity compared to the free chalcone molecule, particularly toward nucleophilic interactions. This increase in chemical softness and electrophilicity highlights the crucial role of metal coordination in modulating the physicochemical properties of the chalcone ligand. The strong correlation between experimental observations and theoretical predictions underscores the robustness of the proposed synthesis and stabilization mechanism. The intrinsic biocompatibility of 2′-hydroxychalcone, combined with its well-established antioxidant, antibacterial, and anticancer properties, renders the synthesized chal–AgNPs highly promising for biomedical and therapeutic applications. The present chalcone-assisted synthesis strategy can be readily extended to the preparation of other noble and transition metal nanoparticles, enabling the design of a wide range of functional nanomaterials with tailored properties. In the future, in-depth *in vitro* and *in vivo* investigations are warranted to further validate the therapeutic potential, biosafety, and pharmacological efficacy of chalcone-capped AgNPs. The integration of advanced computational modeling with experimental approaches may also facilitate the rational design of multifunctional nanoplatforms for targeted drug delivery, imaging, and catalytic applications.

## Conflicts of interest

Authors declare no competing financial interest.

## Supplementary Material

RA-016-D5RA06439B-s001

## Data Availability

All data will be made available as required. Supplementary information (SI) is available. See DOI: https://doi.org/10.1039/d5ra06439b.
