# Supplementary material for: Structural, electronic and bioactive profiling of a 2′-hydroxychalcone–Ag composite: a DFT-supported biomedical study on anticancer, antioxidant and antibacterial activities
Source: RSC Adv. 2026 Feb 16;16(10):9040–53. doi: 10.1039/d5ra06439b (PMC12908004; doi:10.1039/d5ra06439b)
Supplement: RA-016-D5RA06439B-s001 [file RA-016-D5RA06439B-s001.pdf]

Supplementary Information (SI) for RSC Advances.
This journal is © The Royal Society of Chemistry 2026

Supplementary Information

Chalcone-Ag composite for Biomedical Applications: Experimental and DFT investigation

Ayesha Latif Butt[1], Farhat Saira*[2], Safeer Ahmed*[1], Sumbal Tahir[2], Anila Iqbal[2], Asma Siddiqa[2], Kehkashan Mazhar[3], Syeda Sohaila Naz[2]

[1]Department of Chemistry, Quaid-i-Azam University, 45320 Islamabad, Pakistan.

[2]Nanoscience and Technology Division, National Centre for Physics, 44000 Islamabad, Pakistan.

[3]Institute of Biomedical and Genetic Engineering (IBGE), KRL Hospital, G-9 Islamabad, Pakistan.

*fsghaus@gmail.com (Farhat Saira)
safeerad@qau.edu.pk (Safeer Ahmad)

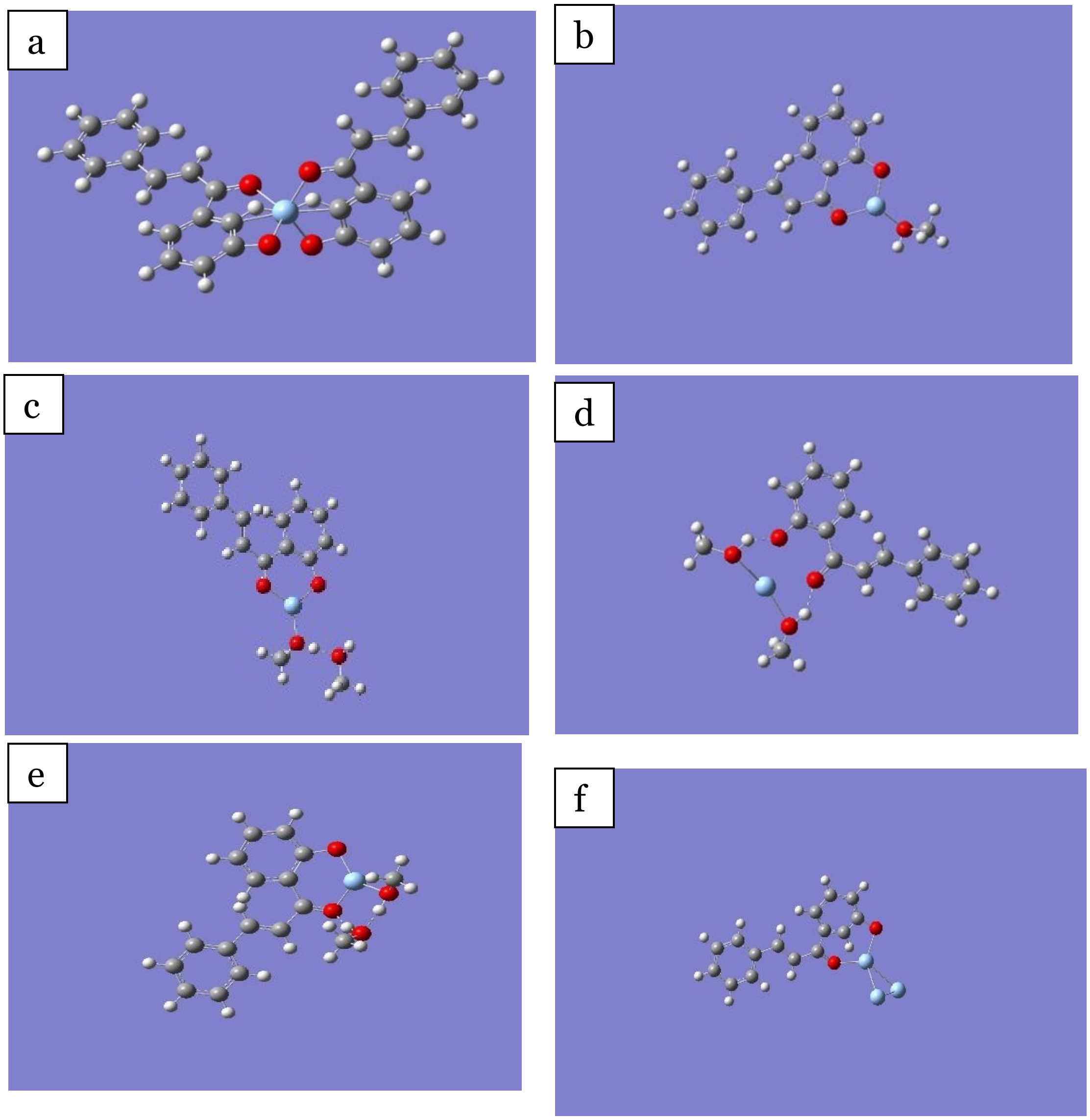
a
b
c
d
e
f

**No.**	**Atom**	**Charge**
**1**	C	-0.118673
**2**	C	0.242607
**3**	C	-0.138401
**4**	C	-0.170577
**5**	C	-0.095364
**6**	C	-0.095338
**7**	H	0.200304
**8**	H	0.157805
**9**	H	0.153546
**10**	H	0.181879
**11**	C	0.193903
**12**	C	-0.204705
**13**	C	-0.129776
**14**	C	-0.072962
**15**	C	-0.032655
**16**	C	-0.171274
**17**	C	-0.161321
**18**	C	-0.119196
**19**	C	-0.170980
**20**	H	0.169388
**21**	H	0.160037
**22**	H	0.155606
**23**	H	0.157410
**24**	H	0.155585
**25**	H	0.193797
**26**	H	0.169686
**27**	O	-0.364605
**28**	O	-0.615770
**29**	H	0.370045

Table S1. Mulliken Atomic Charge on each atom in 2-hydroxychalcone
